# Critical Role for Malic Enzymes in MYC-Mediated Cellular Adaptation to Glutamine Depletion

**DOI:** 10.3390/metabo16040282

**Published:** 2026-04-20

**Authors:** Yufan Si, Wei Li, Yang Chen, Jiayang Yuan, Chenrui Hu, Yanan Liu, Li Li

**Affiliations:** State Key Laboratory of Medical Molecular Biology, Department of Cell Biology, Institute of Basic Medical Sciences Chinese Academy of Medical Sciences, School of Basic Medicine Peking Union Medical College, Beijing 100005, China

**Keywords:** malic enzyme, glutamine starvation, MYC, metabolic adaptation

## Abstract

**Background/Objectives:** MYC-driven tumors exhibit significant glutamine addiction, but the metabolic adaptation mechanisms enabling their survival under glutamine deprivation remain incompletely understood. Malic enzymes catalyze the oxidative decarboxylation of malate to pyruvate while generating NADPH, linking central carbon metabolism to redox homeostasis. This study investigates whether and how ME1 and ME2 mediate cell adaptation to glutamine starvation and explores their functional division in relation to p53 status. **Methods:** Using MYC-amplified, p53-mutant (G266E) SF188 glioblastoma cells, we performed siRNA-mediated knockdown, overexpression, and rescue experiments. Cell survival was assessed by trypan blue exclusion and Annexin V/PI staining. ROS levels and NADP^+^/NADPH ratios were measured by DCFH-DA fluorescence and enzymatic assays. Metabolite tracing was conducted using [U-^13^C_5_] glutamine followed by LC-MS. Key findings were validated in additional cell lines including HCT116, U2OS and MDA-MB-231. **Results:** ME1 and ME2 promote SF188 cell survival under glutamine deprivation, an effect that depends on their catalytic activity but is independent of TCA cycle anaplerosis. ME1 maintains redox balance by generating NADPH, and antioxidant treatment rescues the survival defect caused by ME1 knockdown. In contrast, ME2 does not contribute to redox regulation but stabilizes mutant p53 (G266E) via proteasome inhibition. Both of these pro-survival functions are attenuated upon MYC knockdown, suggesting a dependency on MYC expression. Across all cell lines tested, ME1 and ME2 also promote survival through redox maintenance, although the isoform responsible for antioxidant function differs. **Conclusions:** ME1 and ME2 support metabolic adaptation to glutamine starvation through distinct, isoform-specific mechanisms that depend on MYC expression and p53 mutation status. These findings suggest malic enzymes as potential therapeutic targets in MYC-driven, p53-mutant tumors.

## 1. Introduction

The metabolism in tumor cells is markedly different from that of normal cells in differentiated tissues, especially in the consumption and utilization of glucose and glutamine, the two main nutrients of mammalian cells [1,2,3]. As observed in the 1920s by Otto Warburg, tumor cells consume glucose at high rate and convert a large portion of it to lactate even in the presence of adequate oxygen (aerobic glycolysis or the Warburg effect) [4,5]. Subsequent studies revealed that many tumor cells also avidly consume glutamine, the most abundant amino acid in the plasma [6,7,8]. Although both the consequences and causes are still not completely understood, accumulating evidence suggests that these and other metabolic alterations support anabolic metabolism necessary for tumor cell survival and proliferation, and are activated by mutations in tumor suppressors and proto-oncogenes [9,10,11,12]. The oncogene MYC, whose elevated expression is prevalent in tumor cells, has strong effects on cellular metabolism. It upregulates key metabolic pathways, including glucose uptake, glycolysis, glutaminolysis, fatty acid synthesis, and nucleotide biosynthesis [13]. Notably, cancer cells expressing oncogenic MYC are dependent on glutamine for survival, a phenomenon known as glutamine addiction [14,15]. This addiction has been attributed to the role of glutamine in anaplerosis—replenishing tricarboxylic acid (TCA) cycle intermediates consumed for biosynthesis [16,17], and in the maintenance of the intracellular pool of asparagine to support nucleotide synthesis [18]. However, glutamine deprivation not only leads to metabolite depletion but also triggers significant oxidative stress, as glutamine-derived glutamate is a precursor for glutathione, the major intracellular antioxidant [19,20]. Nevertheless, how MYC-driven tumors cope with the oxidative challenge imposed by glutamine deprivation, and whether other metabolic enzymes participate in this adaptive process are still not well characterized.

In addition to MYC, the tumor suppressor p53 also plays an important role in regulating metabolism, a property that is likely crucial for tumor prevention [21,22,23]. p53 suppresses glucose and glutamine consumption, glycolysis, and the pentose phosphate pathway, while stimulating oxidative phosphorylation [24,25,26,27,28]. p53 is the most frequently mutated gene in human tumors [29]. The vast majority of tumor-derived p53 mutations are missense mutations, which not only lose the tumor suppressive function of wild-type p53, but also acquire oncogenic gain-of-function properties, including metabolic regulation, that further enable tumor formation [30,31]. Given that MYC and mutant p53 frequently coexist and cooperate to promote tumorigenesis, further studies have also shown that the two may mutually affect each other’s levels and activities, suggesting the existence of a complex regulatory network between them [32,33,34]. It is plausible that the state of mutant p53 may profoundly affect the metabolic adaptation of these cells to glutamine deprivation. However, the specific role of mutant p53 in this process remains unclear.

Malic enzymes (MEs) are emerging as critical metabolic nodes that connect TCA cycle metabolism to NADPH production, thereby linking energy metabolism to redox homeostasis [35,36] ME1 is localized in the cytoplasm, while ME2 resides in the mitochondria, and both catalyze the oxidative decarboxylation of malate to pyruvate, generating NADPH in the process [37,38]. Through this activity, malic enzymes are positioned to influence multiple cellular processes, including glutamine metabolism, lipid synthesis, and antioxidant defense [25,39,40,41,42,43]. Previous work has demonstrated that ME1 and ME2 are reciprocally regulated by p53 and play essential roles in cellular senescence [25]. However, whether and how malic enzymes contribute to glutamine addiction and mutant p53 stabilization in MYC-driven cancer cells remain unexplored.

In this study, we investigated the functions of ME1 and ME2 in mediating cellular adaptation to glutamine deprivation using SF188 human glioblastoma cells, which harbor MYC amplification and mutant p53 G266E. We found that ME1 and ME2 promote cell survival under glutamine starvation through distinct mechanisms: ME1 alleviates oxidative stress by maintaining redox balance, while ME2 acts through stabilization of mutant p53 protein. Moreover, by extending our analysis to additional cancer cell lines with different p53 backgrounds, we observed that the isoform responsible for the antioxidant function differs and appears to be influenced by p53 status. These findings uncover previously unrecognized, isoform-specific roles for malic enzymes in the metabolic adaptation of MYC-driven tumors and suggest malic enzymes as potential therapeutic targets.

## 2. Materials and Methods

### 2.1. Antibodies and Reagents

The antibodies against the following proteins/epitopes were purchased from the indicated sources: ME2 (cat# HPA008880, Sigma (St. Louis, MO, USA), 1:1000), ME1 (cat# HPA006493, Sigma-Aldrich, 1:1000), actin (cat# 66009-1-Ig, Proteintech (Rosemont, IL, USA), 1:5000), p53 (DO-1, cat# sc-126, Santa Cruz Biotechnology (Dallas, TX, USA), 1:1000), and MYC (cat# ab32072, Abcam (Cambridge, UK), 1:1000). The following reagents were purchased from Sigma: dimethyl L-malate, N-acetyl cysteine (NAC), 4-Hydroxytamoxifen, oxaloacetic acid, methyl pyruvate, lactic acid, propidium iodide, citrate acid, and dimethyl α-ketoglutarate.

### 2.2. Cell Culture and Cell Survival Assay

Cells were maintained in standard culture conditions without any antibiotic. HCT116 and MDA-MB-231 cells were maintained in Dulbecco’s modified Eagle’s medium (DMEM; catalog no. 11965, Thermo Fisher Scientific (Waltham, MA, USA)), U2OS cells in McCoy’s 5A Medium, and SF188 cells in DMEM supplemented with 2 mM additional L-glutamine. All mediums, if not specifically described, were supplemented with 10% fetal bovine serum (FBS; GEMINI (West Sacramento, CA, USA)). For glutamine starvation experiments, DMEM without glutamine (Cat# 11960, Invitrogen (Carlsbad, CA, USA)) was used. Cell survival was determined using trypan blue or propidium iodide exclusion assays.

### 2.3. siRNA and Stable Overexpression Cell Line Construction

siRNAs for ME1, ME2, MYC, and p53 were purchased from Invitrogen. The sequences were:

5′-CCCUGUGGGUAAUUGUGUGUGAU-3′ (ME1#1),
5′-AUAACAAUCAGGUAGAAUCUGGUCA-3′ (ME1#2),
5′-GUCGACAUUUGCACAUAAATT-3′ (ME2#1),
5′-UAUAGUUGAAGGCUUCAGUAUAUUC-3′ (ME2#2),
5′-ACAGCCCACUGGUCCUCAATT-3′ (MYC),
5′-GCUGUGGGUUGAUUCCACATT-3′ (p53).

siRNA transfection was performed using Lipofectamine RNAiMAX transfection reagent (Invitrogen). Briefly, siRNA and Lipofectamine RNAiMAX were diluted in Opti-MEM medium and mixed to form transfection complexes. The complexes were added to cells cultured in complete DMEM. For glutamine deprivation experiments, the medium was removed 24 h after transfection and replaced with glutamine-free DMEM for subsequent culture.

Stable cell lines overexpressing ME1, ME2, or p53 mutants were generated by lentiviral transduction. Lentivirus was produced in HEK293T cells co-transfected with the pBabe construct and packaging plasmids. The viral supernatant was used to infect target cells in the presence of polybrene. Stable transductants were selected with puromycin.

### 2.4. qRT-PCR

Total RNA was extracted using TRIzol (Invitrogen) (Carlsbad, CA, USA) and reverse-transcribed into cDNA using a First-strand cDNA Synthesis System (Marligen Biosciences) (Ijamsville, MD, USA). qRT-PCR was performed using SYBR Green PCR Master Mix (Applied Biosystems) (Waltham, MA, USA) on a 7900HT Fast Real-Time PCR System. The thermal cycling conditions were: 50 °C for 2 min, 95 °C for 10 min, followed by 45 cycles of 95 °C for 15 s and 60 °C for 1 min, and a dissociation curve. Gene expression levels were normalized to ACTB. The primer sequences were as follows: ACTB forward 5′-GACCTGACTGACTACCTCATGAAGAT-3′ and reverse 5′-GTCACACTTCATGATGGAGTTGAAGG-3′; TP53 forward 5′-CACGAGCTGCCCCCAGG-3′ and reverse 5′-TCAGTCGACGTCTGAGT-3′.

### 2.5. Measurements of Metabolites and Glutaminolytic Flux

Levels of glucose and glutamine were determined using YSI 7100 Multiparameter Bioanalytical System (YSI Life Sciences) (Yellow Springs, OH, USA). Levels of citrate were determined using quantification assay kits (BioVision) (Milpitas, CA, USA) or liquid chromatography-mass spectrometry (LC-MS). Flux analysis was performed on TSQ Quantiva Triple Quadrupole mass spectrometer (Thermo, (Waltham, MA, USA)with positive/negative ion switching. MRM mode was used for data acquisition. Mobile phase A was prepared by adding 2.376 mL tributylamine and 0.858 mL acetic acid to HPLC-grade water, then adding HPLC-grade water to 1 L volume. Mobile phase B was HPLC-grade methanol. Synergi Hydro-RP 100A column was used for polar metabolites separation with column temperature at 35 °C. The measured mass isotopomer distributions were corrected for natural enrichments.

The glutaminolytic flux through malic enzymes was calculated from the equation: FME = FP × (Pm + 3)/(Mm + 4), in which FME = malic enzyme flux, FP = total pyruvate flux, Pm + 3 = fraction of pyruvate enriched in all three carbons, and Mm + 4 = fraction of malate enriched in all four carbons.

### 2.6. Measurements of ROS and NADPH

Cells were incubated at 37 °C for 30 min in PBS containing 10 μM 2′,7′-dichlorodihydrofluorescein diacetate (H2-DCFDA, Sigma(St. Louis, MO, USA)). Afterwards, the cells were washed twice in PBS, detached with trypsin, and resuspended in PBS. Fluorescence was immediately measured using a FACS Flow Cytometer (Becton Dickinson (Franklin Lakes, NJ, USA)). The levels of NADPH and NADP+ in culture cells were determined using a NADP+/NADPH Quantification Kit (BioVision (Milpitas, CA, USA)) according to the manufacturer’s instructions.

### 2.7. Western Blotting

Whole-cell lysates were obtained using modified RIPA lysis buffer Yeasen (Shanghai, China) (10 mM Tris_HCl at pH 7.5, 5 mM EDTA, 150 mM NaCl, 1% NP-40, 1% sodium deoxycholate, 0.025% SDS, and complete protease cocktail). Cells were washed and incubated in lysis buffer for 15 min on ice, and boiled in 2× loading buffer. Protein samples were resolved by SDS-PAGE and transferred onto nitrocellulose membrane. The membrane was then blocked in 5% skimmed milk in TBST and probed with the indicated antibodies.

### 2.8. Apoptosis Assay

Apoptosis was assessed using an Annexin V-FITC/PI apoptosis detection kit (Yeasen (Shanghai, China), Cat# 40302ES) according to the instructions. Briefly, cells were harvested, washed with PBS, and resuspended in binding buffer. Annexin V-FITC and propidium iodide were added, and the cells were incubated for 15 min at room temperature in the dark. Stained cells were analyzed by flow cytometry.

### 2.9. Statistical Analysis

All experiments in this work were performed at least three times independently. Statistical significance was analyzed by Student’s *t*-test and expressed as a *p* value. * *p* < 0.05, ** *p* < 0.01, *** *p* < 0.001.

## 3. Results

### 3.1. ME1 and ME2 Regulate Cellular Adaption to Glutamine Deprivation

**Figure 1:**
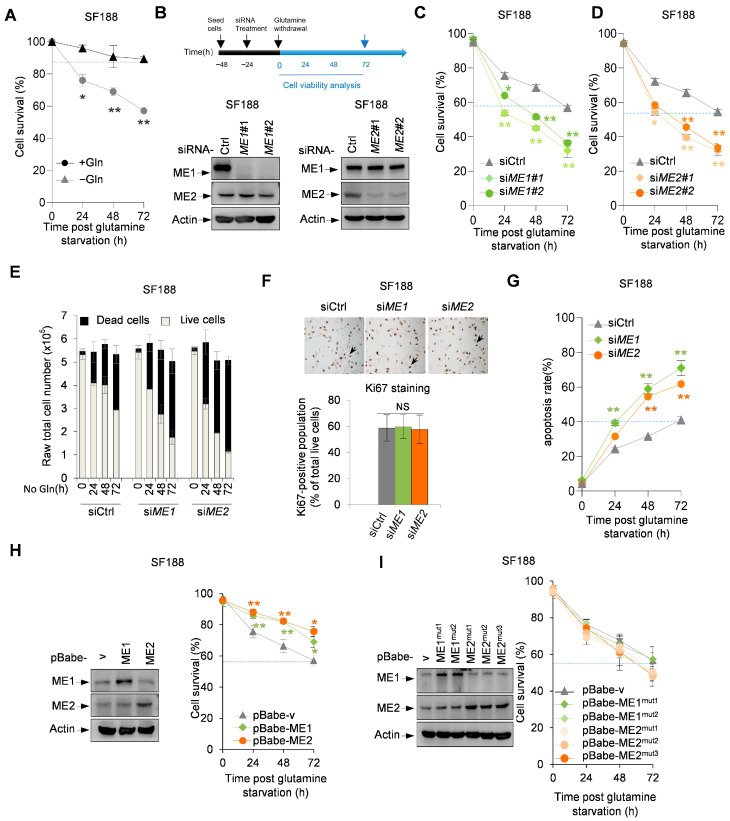
Roles of ME1 and ME2 in regulating cellular adaptation to glutamine depletion. (**A**) Cell survival of SF188 cells cultured in normal or glutamine-deprived medium for 72 h (**B**) Schematic timeline of siRNA transfection and glutamine starvation (**top**) and knockdown efficiency of ME1 and ME2 by two independent siRNAs confirmed by Western blot (bottom; quantified in Appendix A, Table A1). (**C**,**D**) Viability of SF188 cells transfected with control siRNA (siCtrl) or siRNA against ME1 (**C**) or ME2 (**D**) and cultured in glutamine-free medium. (**E**,**F**) Quantification of total live and dead cells (**E**) and KI67 staining (**F**) of SF188 cells transfected indicated siRNAs. Arrows in (F) indicate KI67-positive brown dots. (**H**) protein expression (**left**) and Viability (**right**) of SF188 cells stably expressing wild-type ME1 or ME2 and cultured in glutamine-free medium. (**I**) Viability (**right**) and protein expression (**left**) of SF188 cells stably expressing mutant malic enzymes (ME1mut1, ME1mut2, ME2mut1, ME2mut2, ME2mut3) or vector control and cultured in glutamine-free medium. Data in (**A**), (**C**–**I**) are presented as mean ± SD from three independent experiments. Statistical significance was determined by two-tailed unpaired *t*-test. * *p* < 0.05, ** *p* < 0.01, no annotation is shown in the figures for ns (no statistical significance).

In order to investigate the role of MEs in the adaptation of tumor cells to glutamine depletion, we used SF188 human glioblastoma cells, which express MYC at oncogenic levels and are dependent on glutamine for survival. We first confirmed the response of SF188 cells to glutamine deprivation. Cells cultured in glutamine-deprived medium exhibited significantly reduced viability compared to those grown under normal conditions, confirming that glutamine is essential for SF188 cell survival (Figure 1A). Surprisingly, knocking down ME1 or ME2 by two independent siRNAs (Figure 1B) strongly reduced the survival of SF188 cells under this metabolic stress condition (Figure 1C,D). Glutamine deprivation stopped the proliferation of control cells as effectively as that of ME1/2-depleted cells (Figure 1E,F), ruling out distinct proliferation rates as a potential reason for different cell death observed in these cells. Annexin V/PI staining further revealed that ME1 or ME2 knockdown significantly increased the percentages of both early and late apoptotic cells within 72 h of glutamine deprivation, whereas control cells maintained low levels of apoptosis (Figure 1G).

Conversely, forced expression of ME1 or ME2 significantly improved the survival of SF188 cells in glutamine-free medium (Figure 1H). To evaluate whether this effect was due to the catalytic activity of malic enzymes, we tested two inactive ME1 mutants (ME1^mut1^ and ME1^mut2^) and three inactive ME2 mutants (ME2^mut1^, ME2^mut2^, and ME2^mut3^) [25]. None of these mutants were able to protect SF188 cells from glutamine-deprivation-induced apoptosis (Figure 1G). These results suggest that ME1 and ME2 promote the survival of SF188 cells in glutamine-deprived medium, and this function of malic enzymes is attributed to their enzymatic activity.

### 3.2. The Effects of ME1 and ME2 on Cell Survival Are Likely Independent of Anaplerosis

Glutamine is an important anaplerotic substrate for the replenishment of the TCA intermediates used for biosynthesis [44,45]. MYC-driven tumor cells grown in glutamine-free medium can be rescued from apoptosis by the addition of TCA-cycle intermediates such as oxaloacetate (OAA) or the TCA cycle substrate pyruvate [14,15]. To address the mechanism by which ME1/2 modulate SF188 cell survival under glutamine starvation, we investigated the effects of malic enzymes on TCA cycle-associated metabolites.

**Figure 2:**
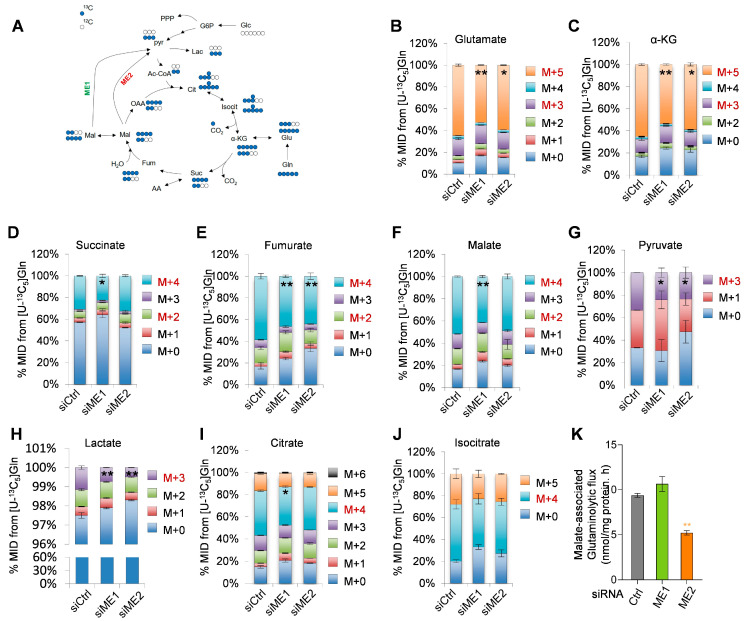
Effects of MEs on glutamine catabolism. (**A**–**I**) Mass isotopomer distributions of indicated metabolites (glutamate, α-ketoglutarate, succinate, fumarate, malate, pyruvate, lactate, citrate, isocitrate) in SF188 cells transfected with control, ME1, or ME2 siRNA for 48 h and then cultured for 12 h in medium containing [U-^13^C_5_]-glutamine. The relative abundance of each mass isotopomer was determined by LC-MS and is denoted as m + n, where n is the number of ^13^C atoms. (**K**) Malic enzyme-associated glutaminolytic flux from malate to pyruvate. Data in (**A**–**K**) are presented as mean ± SEM from four biological replicates per condition. Statistical significance was determined by two-tailed unpaired *t*-test only for fully labeled isotopologues. * *p* < 0.05, ** *p* < 0.01.

We cultured SF188 cells in medium containing [U-13C5] glutamine (glutamine labeled with 13C at all five positions) and measured the levels of glutamine-derived, TCA cycle-associated metabolites to examine the role of malic enzymes in glutamine catabolism (Figure 2A). Silencing ME1 or ME2 led to reduced levels of several metabolites, including glutamate (m + 5), α-ketoglutarate (α-KG, m + 5), fumarate (m + 4), pyruvate (m + 3), and lactate (m + 3) (Figure 2B,C,E,G,H). Silencing ME1 also decreased succinate (m + 4), malate (m + 4), and citrate (m + 4), while silencing ME2 had only a limited impact on these metabolites(Figure 2D,F,I,J). Notably, ME1 knockdown showed a minimal effect on glutaminolytic flux from malate to pyruvate, while inhibition of ME2 reduced glutaminolytic flux by ~40% (Figure 2K). Collectively, these results suggest that both ME1 and ME2 influence glutamine metabolism, with both similar and distinct effects.

**Figure 3:**
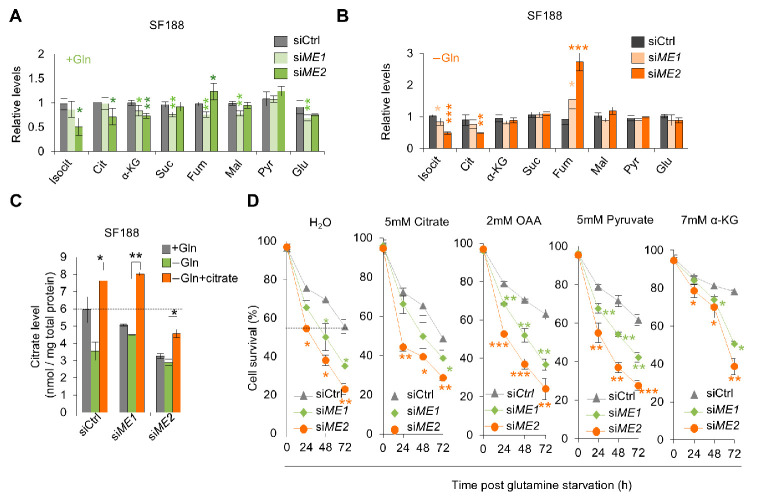
The effects of ME1 and ME2 on cell survival are likely independent of anaplerosis. (**A**,**B**) Intracellular levels of indicated metabolites in SF188 cells transfected with control, ME1, or ME2 siRNA and cultured in complete medium (**A**) or glutamine-free medium (**B**) for 12 h. Metabolites were quantified by LC-MS and normalized to protein content. Isocit, isocitrate; Cit, citrate; α-KG, α-ketoglutarate; Suc, succinate; Fum, fumarate; Mal, malate; Pyr, pyruvate; Glu, glutamate. (**C**) Intracellular citrate levels in SF188 cells transfected with control, ME1, or ME2 siRNA for 24 h and then cultured in complete or glutamine-free medium with or without citrate for another 24 h. (**D**) Cell survival of SF188 cells transfected with control, ME1, or ME2 siRNA and cultured in glutamine-free medium supplemented with H_2_O, citrate (5 mM), OAA (2 mM), methyl-pyruvate (5 mM), or dimethyl α-ketoglutarate (7 mM) as indicated. Data in (**A**–**D**) are presented as mean ± SD from three independent experiments. Statistical significance was determined by two-tailed unpaired *t*-test. * *p* < 0.05, ** *p* < 0.01, *** *p* < 0.001.

We next sought to determine how ME1 and ME2 influence TCA cycle metabolite profiles under different nutrient conditions. When cultured in complete medium, both ME1- and ME2-depleted cells showed a significant reduction in the levels of α-KG. ME1-depleted cells also contained reduced levels of succinate, fumarate, malate, and glutamate, while ME2-depleted cells contained reduced levels of isocitrate and citrate. The levels of pyruvate were minimally affected in cells devoid of either ME1 or ME2 (Figure 3A). When grown in the absence of glutamine, ME1- and especially ME2-depleted cells showed reduced levels of total citrate and isocitrate, but elevated levels of total fumarate. In contrast, these cells exhibited minimal changes in the levels of α-KG, succinate, malate, pyruvate, and glutamate (Figure 3B).

Given that ME1 and ME2 are positioned at the interface between the TCA cycle and glutaminolysis, we asked whether their pro-survival function under glutamine starvation is mediated by maintaining TCA cycle intermediates. However, addition of exogenous membrane-permeable citrate, which restored cellular citrate levels (Figure 3C), failed to rescue ME1/2-depleted SF188 cells from glutamine-induced cell death (Figure 3D). Addition of OAA, pyruvate or α-KG improved survival of control cells, but it did not diminish the difference between ME1/2-depleted cells and control cells (Figure 3D). These findings suggest that the reduction in the survival of ME1/2-depleted SF188 cells grown in glutamine-free medium is unlikely due to the alterations in the levels of TCA cycle metabolites in these cells.

### 3.3. ME1 Promotes the Survival of SF188 Cells by Enhancing Anti-Oxidant Defense

**Figure 4:**
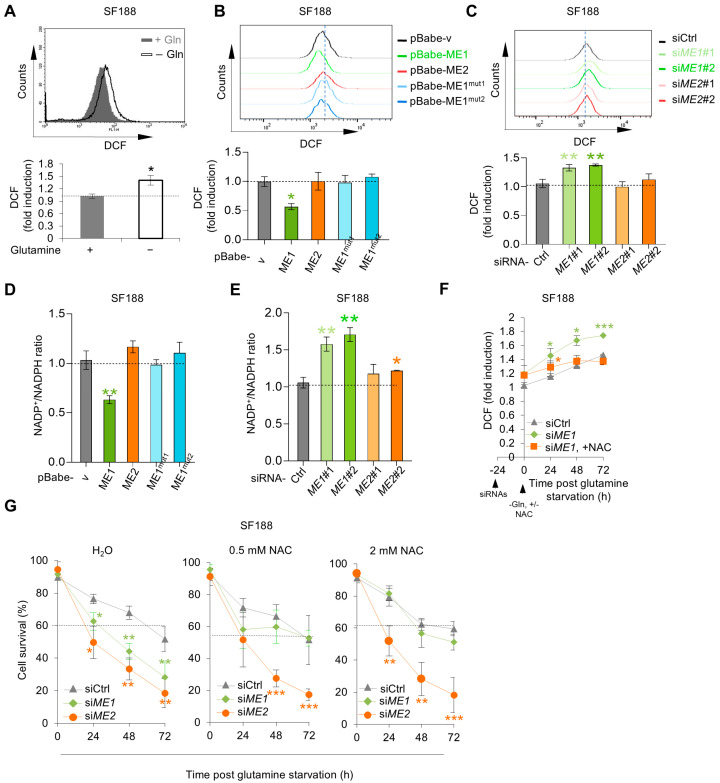
Role of ME1 in anti-oxidant defense in SF188 cells. (**A**) ROS levels in SF188 cells cultured in complete or glutamine-free medium, measured by DCF staining. (**B**,**C**) ROS levels in SF188 cells stably expressing ME1, ME2, or vector control (**B**), or transfected with indicated siRNAs (**C**), determined by FACS. (**D**,**E**) NADP^+^/NADPH ratio in SF188 cells stably expressing ME1, ME2, or vector control (**D**), or transfected with indicated siRNAs (**E**). (**F**) ROS levels in SF188 cells transfected with control or ME1 siRNA and cultured in glutamine-free medium with or without NAC. (**G**) Cell survival of SF188 cells transfected with control, ME1, ME2 siRNA and cultured in glutamine-free medium containing indicated concentrations of NAC. Data in (**A**–**G**) are presented as mean ± SD from three independent experiments. Statistical significance was determined by two-tailed unpaired *t*-test. * *p* < 0.05, ** *p* < 0.01, *** *p* < 0.001.

To investigate the mechanisms by which malic enzymes promote the survival of SF188 cells in glutamine-free medium, we noticed that glutamine deprivation significantly increased ROS levels in these cells (Figure 4A). ME1 and ME2 are important for NADPH production and redox homeostasis [25,44,45], raising the possibility that ME1 and/or ME2 may counter the rise in ROS in glutamine-deprived SF188 cells. Indeed, forced expression of ME1 strongly reduced ROS and the NADP+/NADPH under this condition (Figure 4B,D), while depletion of ME1 significantly increased ROS levels and the NADP+/NADPH ratio in these cells (Figure 4C,E). We therefore investigated whether the elevated ROS levels contribute to the reduced survival of ME1-depleted cells under glutamine deprivation by treating them with the antioxidant N-acetylcysteine (NAC). Addition of the NAC effectively reduced cellular ROS levels in ME1-depleted cells (Figure 4F), and promoted the survival of ME1-depleted cells in glutamine-free medium in a dose-dependent manner (Figure 4G). At a concentration of 2 mM, NAC almost completely restored the survival of ME1-depleted cells to levels seen in control cells (Figure 4G). Compared to ME1, ME2 has less of an effect on the abundance of ROS in SF188 cells (Figure 4B,C). Consistently, NAC minimally influenced the survival of ME2-depleted cells upon glutamine deprivation (Figure 4G). These results suggest that ME1, but not ME2, protects SF188 cells from glutam ine-deprivation-induced cell death by enhancing anti-oxidant defense.

### 3.4. ME2 Stabilizes p53 G266E to Support Cell Survival During Glutamine Starvation

**Figure 5:**
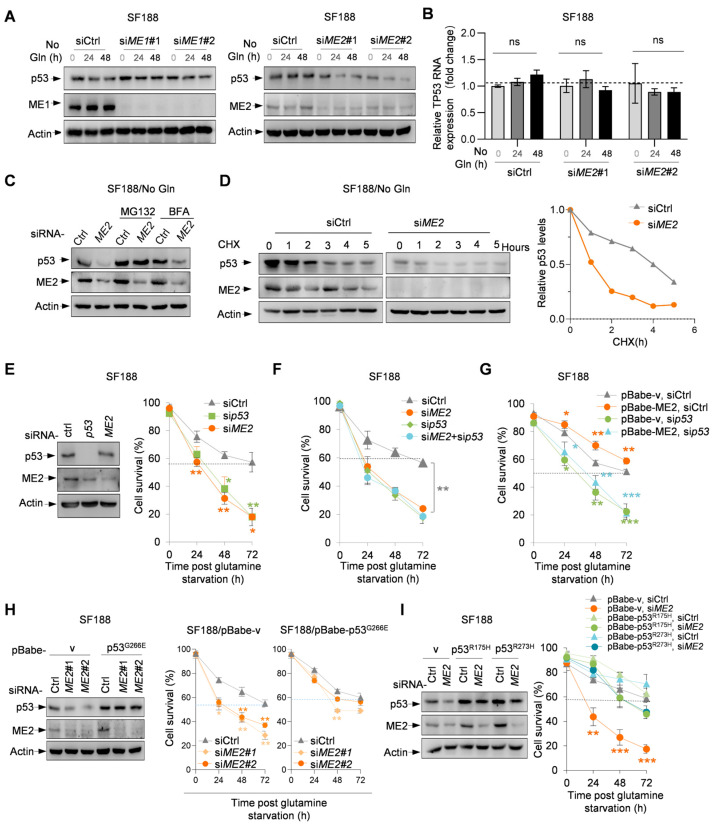
ME2 enhances the survival of SF188 cells by maintaining the levels of p53 G266E in glutamine-deprived medium. (**A**,**B**) SF188 cells transfected with control, ME1, or ME2 siRNA were cultured in glutamine-free medium for 0, 24, or 48 h. Western blot analysis of p53 G266E protein expression (**A**) and qRT-PCR analysis of TP53 mRNA levels (**B**). (**C**) SF188 cells transfected with control or ME2 siRNA were treated with or without MG132 (10 μM) or BFA (5 μg/mL) for 6 h. p53 G266E protein levels were analyzed by Western blot. (**D**) SF188 cells transfected with control or ME2 siRNA were treated with cycloheximide (CHX, 100 μg/mL) for the indicated times. p53 G266E protein levels were analyzed by Western blot; relative p53/actin ratios are shown. (**E**,**F**) Protein expression (**left**) and cell survival (**right**) of SF188 cells transfected with control, ME2, or p53 siRNA and cultured in glutamine-free medium. (**G**) Cell survival of SF188 cells stably expressing ME2 or vector control, treated with control or p53 siRNA and cultured in glutamine-free medium. (**H**,**I**) Protein expression (**left**) and cell survival (**right**) of SF188 cells stably expressing indicated p53 mutants or vector control, transfected with control or ME2 siRNA and cultured in glutamine-free medium. Data in (**B**), (**E**–**I**) are presented as mean ± SD from three independent experiments. Statistical significance was determined by two-tailed unpaired *t*-test. * *p* < 0.05, ** *p* < 0.01, *** *p* < 0.001, no annotation is shown in the figures for ns (no statistical significance).

In addition to MYC over-expression, SF188 cells harbor a p53 mutation, Gly266-to-Glu (G266E) [46]. Tumor-derived p53 missense mutations, including G266E, often acquire gain-of-function properties that enhance tumor survival and proliferation. Previous studies have shown that ME2 stabilizes mutant p53 through its downstream metabolite 2-hydroxyglutarate (2-HG). We next asked whether this regulatory axis operates under glutamine-deprived conditions. Of note, silencing ME2 led to a strong reduction in the protein levels of the endogenous p53 G266E, while silencing ME1 had a minimal effect (Figure 5A). ME2 knockdown did not alter TP53 mRNA levels in SF188 cells (Figure 5B), indicating that ME2 regulates mutant p53 at the post-transcriptional level. Treatment with the proteasome inhibitor MG132 restored p53 G266E protein levels in ME2-depleted cells (Figure 5C), and CHX chase assay revealed accelerated p53 degradation upon ME2 knockdown (Figure 5D). These data demonstrate that ME2 stabilizes mutant p53 by inhibiting its proteasome-mediated degradation under glutamine deprivation.

Interestingly, silencing p53 G266E markedly reduced the survival of SF188 cells grown in glutamine-free medium, similar to the effect of silencing ME2 (Figure 5E). In cells devoid of p53 G266E, knockdown of ME2 did not further decrease cell survival (Figure 5F), while forced expression of ME2 did not enhance it (Figure 5G). Conversely, when the expression of p53 G266E was elevated in SF188 cells, the effect of ME2 knockdown on both the p53 protein levels and cell survival was significantly attenuated (Figure 5H). These results demonstrated that p53 G266E is critical for the survival of SF188 cells in glutamine-deprived medium and that ME2 enhances the survival of SF188 cells by maintaining the levels of p53 G266E. To test whether other tumor-derived p53 mutants, like p53 G266E, can promote the survival of SF188 cells and negate the requirement of ME2, we used two “hot-spot” p53 mutations, R175H and R273H. Expression of each mutation restored the survival of ME2-depleted cells to levels observed for the control cells (Figure 5G,H). Thus, other tumor-associated p53 mutants tested in this study similarly supported SF188 cell survival under glutamine deprivation, suggesting that this ME2-mediated survival mechanism may extend to a broader range of p53 mutants.

### 3.5. Dependence of Malic Enzymes’ Role on MYC in SF188 Cells

**Figure 6:**
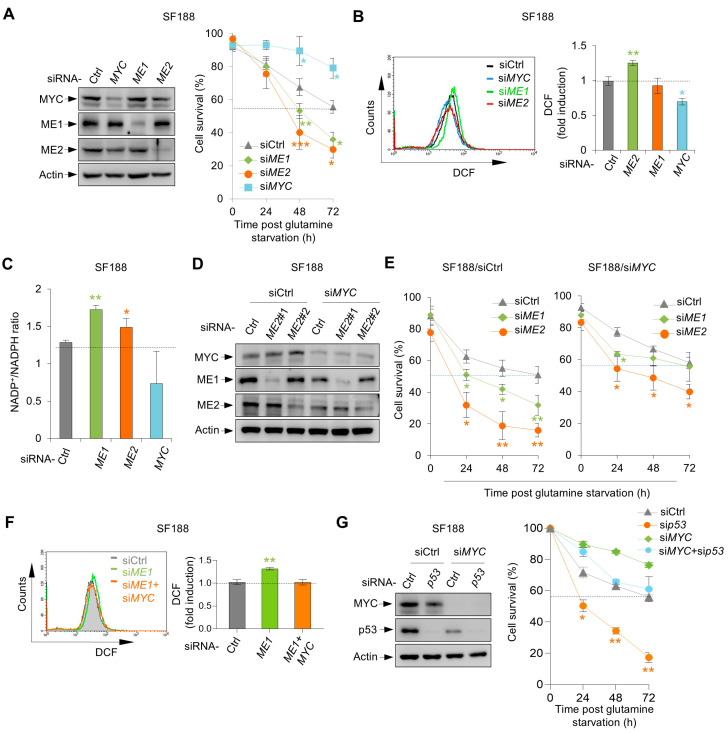
The pro-survival roles of malic enzymes in SF188 are related to the expression of MYC. (**A**–**C**) SF188 cells were transfected with control, ME1, ME2, or MYC siRNA and cultured in glutamine-free medium. (**A**) Protein expression (left) and cell survival (right); (**B**) ROS levels measured by DCF staining; (**C**) NADP^+^/NADPH ratio. (**D**,**E**) SF188 cells were sequentially transfected with indicated siRNAs and cultured in glutamine-free medium. Western blot analysis of protein expression (**D**) and cell survival (**E**). (**F**) ROS levels in SF188 cells transfected with indicated siRNAs, measured by DCF staining. (**G**) Protein expression (left) and cell survival (right) of SF188 cells transfected with control or MYC siRNA, and further transfected with control or p53 siRNA, then cultured in glutamine-free medium. Data in (**A**–**G**) are presented as mean ± SD from three independent experiments. Statistical significance was determined by two-tailed unpaired *t*-test. * *p* < 0.05, ** *p* < 0.01, *** *p* < 0.001.

The addiction of SF188 cells to glutamine is due to the expression of MYC at oncogenic levels [14]. As expected, knockdown of MYC by siRNA significantly enhanced cell survival under glutamine deprivation (Figure 6A). Consistent with a role for MYC in the promotion of oxidative stress, knocking down MYC reduced both ROS levels and the NADP^+^/NADPH ratio in SF188 cells (Figure 6B,C). Of note, in MYC-knockdown cells, silencing ME1 had no effect on survival, especially upon prolonged glutamine deprivation (at 72 h) (Figure 6D,E). Furthermore, in MYC-knockdown cells, ME1 knockdown failed to elevate ROS levels (Figure 6F). These results suggest that ME1 may counter the pro-oxidative effect of MYC in SF188 cells.

Silencing MYC also significantly restored the survival of ME2-depleted SF188 cells (Figure 6E). Because ME2 stabilizes p53 G266E, we next examined whether the role of p53 G266E in cell survival is related to MYC. Compared to control cells, cells devoid of MYC were much less sensitive to p53 knockdown (Figure 6G). This result suggests that MYC expression renders SF188 cells highly dependent on p53 G266E, hence ME2, for survival under glutamine deprivation. Together, these results indicate that the pro-survival roles of malic enzymes in SF188 are related to the expression of MYC.

### 3.6. Validation of the p53-Dependent Functional Switch of ME1 and ME2 in Multiple Cell Lines

To further test whether the functional division of ME1 and ME2 observed in SF188 cells represents a general phenomenon, we extended our analysis to several additional cell lines with defined p53 and MYC backgrounds.

**Figure 7:**
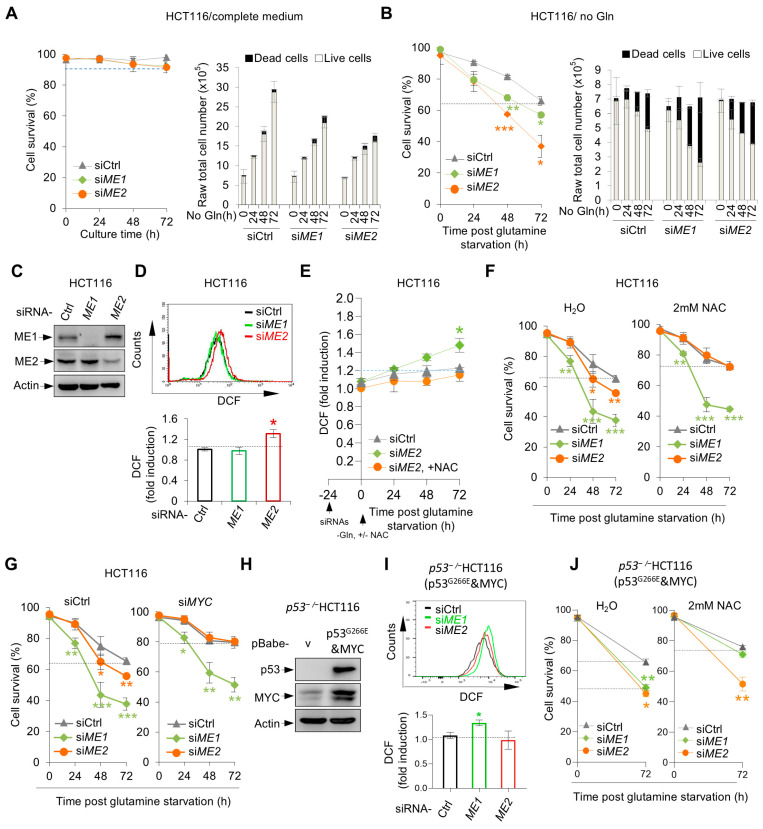
ME1 and ME2 promote cell survival under glutamine deprivation in HCT116. (**A**,**B**) HCT116 cells were transfected with control, ME1, or ME2 siRNA and cultured in complete medium (**A**) or glutamine-free medium (**B**) for 72 h. Cell survival and raw total cell number are shown. (**C**,**D**) HCT116 cells were transfected with indicated siRNA. (**C**) Western blot analysis of protein expression; (**D**) ROS levels measured by DCF staining. (**E**,**F**) HCT116 cells transfected with indicated siRNA were cultured in glutamine-free medium with or without 2 mM NAC. ROS levels (**E**) and cell survival (**F**) are shown. (**G**) HCT116 cells were sequentially transfected with control or MYC siRNA, followed by control, ME1, or ME2 siRNA, then cultured in glutamine-free medium. Cell survival is shown. (**H**–**J**) p53-knockout HCT116 cells (p53^−^/^−^) stably expressing vector control or p53 G266E were transfected with control, ME1, or ME2 siRNA and cultured in glutamine-free medium. (**H**) Western blot analysis of protein expression; (**I**) ROS levels; (**J**) cell survival. Data in (**A**,**B**,**D**–**J**) are presented as mean ± SD from three independent experiments. Statistical significance was determined by two-tailed unpaired *t*-test. * *p* < 0.05, ** *p* < 0.01, *** *p* < 0.001.

First, we examined HCT116 colorectal cancer cells, which express high levels of MYC but are p53-wild-type. Knockdown of ME1 or ME2 had no effect in complete medium (Figure 7A) but significantly reduced cell survival under glutamine deprivation (Figure 7B), indicating that ME1/2 dependency is conserved in another MYC-high background.

We next assessed the functional division of ME1 and ME2 in HCT116 cells. Knockdown of ME2, but not ME1, led to a marked increase in ROS levels (Figure 7D). Importantly, the antioxidant NAC fully rescued both the ROS accumulation and the survival defect caused by ME2 knockdown, whereas it had no effect on the cell death induced by ME1 knockdown(Figure 7E,F). Similarly, upon MYC knockdown, the survival defect caused by ME2 depletion was fully rescued, but ME1 depletion still impaired cell survival (Figure 7G). These data confirm that malic enzymes promote cell survival under glutamine deprivation by maintaining redox homeostasis. Notably, in SF188 cells (p53-mutant) the antioxidant function is carried out by ME1, whereas in HCT116 cells (p53-wild-type) it is performed by ME2. To investigate whether this difference is related to the distinct p53 mutation status between the two cell lines, we reconstituted p53-kncokout HCT116 cells (p53^−/−^HCT116) with the p53 G266E mutant (Figure 7H). In p53 G266E-expressing HCT116 cells, the functional division switched: ME1 gained the ability to suppress ROS (ME1 knockdown increased ROS, which was rescued by NAC), while the antioxidant function of ME2 was markedly reduced (Figure 7I,J). These results indicate that the presence of mutant p53 can influence the antioxidant division of ME1 and ME2.

**Figure A1:**
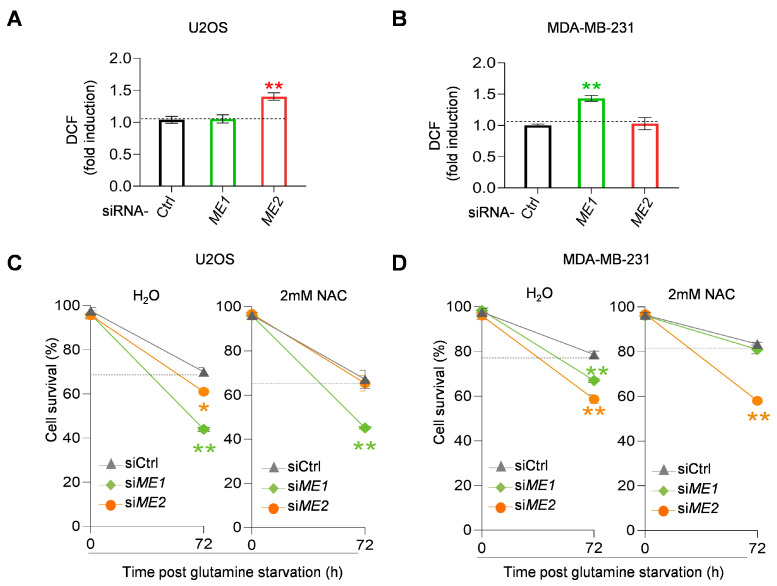
NAC reverses survival defect in ME2- or ME1-depleted cells under glutamine deprivation. (**A**,**B**) ROS levels after transfection with control, ME1, or ME2 siRNA in U2OS cells (**A**) and MDA-MB-231 cells (**B**). (**C**,**D**) Cell survival after transfection with control, ME1, or ME2 siRNA, treated with or without 2 mM NAC, in U2OS cells (**C**) and MDA-MB-231 cells (**D**). Data in (**A**–**D**) are presented as mean ± SD from three independent experiments. Statistical significance was determined by two-tailed unpaired *t*-test. * *p* < 0.05, ** *p* < 0.01, no annotation is shown in the figures for ns (no statistical significance).

Finally, we analyzed two additional cell lines: p53-wild-type U2OS osteosarcoma cells and p53-mutant MDA-MB-231 breast cancer cells. In U2OS, ME2 was the major antioxidant isoform, and NAC treatment fully rescued the survival defect upon ME2 knockdown. Conversely, in MDA-MB-231, ME1 played the dominant antioxidant role, and its knockdown-induced survival defect was fully rescued by NAC (Appendix A, Figure A1). Together, evidence from these three independent cell lines lead to the conclusion that: in the context of high MYC expression, ME1 and ME2 promote cell survival under glutamine deprivation through distinct mechanisms, and the specific functional division is influenced by cellular context, including p53 status. In p53-mutant cells, ME1 assumes the antioxidant function, whereas ME2 shifts to stabilizing mutant p53 to promote survival.

## 4. Discussion

Malic enzymes ME1 and ME2 are emerging as critical metabolic nodes that connect TCA cycle metabolism to NADPH production and redox homeostasis. In this study, we uncover previously unrecognized, isoform-specific roles for ME1 and ME2 in enabling MYC-driven glioblastoma cells to survive glutamine deprivation. While both enzymes catalyzing the same biochemical reaction—the oxidative decarboxylation of malate to pyruvate, they promote survival through fundamentally different pathways. ME1 knockdown elevates ROS levels and increases the NADP+/NADPH ratio, and the resulting survival defect is completely rescued by the antioxidant NAC. These observations establish ME1 as a key regulator of redox homeostasis under glutamine limitation, consistent with its cytosolic localization and its role in supplying NADPH for glutathione recycling and other antioxidant systems. In contrast, ME2 knockdown has minimal impact on ROS levels and is not rescued by NAC, but instead leads to a marked reduction in mutant p53 G266E protein levels. Genetic epistasis experiments confirm that ME2 acts upstream of mutant p53, and the ability of other tumor-derived p53 mutants (R175H, R273H) to substitute for G266E suggests that ME2-mediated stabilization of mutant p53 may be a general feature of p53-mutant, MYC-driven tumors.

This functional divergence between ME1 and ME2 likely reflects their distinct subcellular localizations—ME1 in the cytoplasm and ME2 in the mitochondria—which expose them to different metabolic pools and protein interaction networks. Mitochondrial ME2 may be positioned to interact directly with p53, which has been reported to localize to mitochondria under certain stress conditions [46,47,48]. Furthermore, the relative contributions of ME1 and ME2 to NADPH production and antioxidant defense exhibit cell type-specific patterns. In several tumor and normal cell lines that were previously tested (e.g., IMR90, U2OS, and HCT116), ME2 plays a more profound role in NADPH production and ROS detoxification than ME1 [25]. By contrast, in human pancreatic ductal adenocarcinoma (PDAC) cells as well as in SF188 and MDA-MB-231 cells, ME1 plays an important role in NADPH production and redox regulation [44]. One explanation is that the availability of the related metabolic enzymes may influence the relative contribution of ME1 and ME2 to anti-oxidant defense. Moreover, our analysis across multiple cell lines revealed that p53 mutation status also influences this functional division.

Both ME1- and ME2-mediated survival mechanisms are dependent on MYC expression, as MYC knockdown abrogates the phenotypes induced by ME depletion. This is consistent with the well-established role of MYC in driving glutamine addiction and oxidative stress. MYC-driven tumors face higher oxidative stress due to increased mitochondrial biogenesis and metabolic activity, which may make them particularly dependent on ME1-mediated ROS detoxification. Consistent with this, we observed that MYC knockdown rendered SF188 cells much less sensitive to p53 knockdown, indicating that high MYC expression drives a dependency on mutant p53 for survival. Previous studies have shown that mutant p53 can transactivate MYC expression, and reciprocally, MYC can stabilize mutant p53 through the mevalonate pathway or directly binding to its promoter to enhance p53 transcription [32,34,49,50], suggesting a positive feedback loop that reinforces this dependency. Consequently, the reliance on mutant p53 oncogenic functions may make these cells parallelly dependent on ME2-mediated stabilization of mutant p53.

Nevertheless, MYC coordinates a metabolic and oncogenic environment that makes cancer cells vulnerable to disruption of ME1 or ME2. This dependency conforms to the “synthetic lethality”: when an oncogene (such as MYC) is activated, cells simultaneously rely on certain metabolic enzymes for survival, which are non-essential or functionally redundant in normal cells. Previous studies have confirmed that MYC-driven tumors are sensitive to glutaminase GLS1 inhibitors [51,52], and our findings suggest that malic enzymes represent another class of synthetic lethal targets. It should be noted that while our data demonstrate that MYC knockdown attenuates ME1/2-mediated survival, the precise dose-dependency and whether MYC re-expression suffices to restore these functions remain to be determined in future studies using inducible systems.

Given that the catalytic activity of ME1 and ME2 is critical for their pro-survival functions, the development of specific small-molecule inhibitors represents a direct therapeutic strategy. Preliminary efforts have been made to develop ME2 inhibitors, but their selectivity and pharmacokinetic properties remain to be optimized. Considering the functional differences between ME1 and ME2 across distinct cell types, combination strategies targeting both enzymes, or selective targeting of ME2 based on tumor p53 status, may achieve enhanced therapeutic efficacy. For p53-mutated MYC-driven tumors, ME2 inhibitors may be the preferred strategy, In contrast, for tumor types where ME1 function predominates (e.g., pancreatic cancer), targeting of ME1 may be more appropriate. Furthermore, since ME2 knockdown concurrently activates wild-type p53 and impairs the stability of mutant p53, ME2 inhibitors may exert distinct therapeutic effects in p53 wild-type versus mutant tumors, and this needs to be distinguished in preclinical studies.

## Data Availability

The data presented in this study are available in the main article.

## Appendix A

**Table A1:** Knockdown efficiency of siRNAs used in this study.

**siRNA Target**	**Knockdown Efficiency (%)**	**Corresponding Figure**
ME1#1	79	Figure 1B
ME1#2	86	Figure 1B
ME2#1	72	Figure 1B
ME2#2	69	Figure 1B
p53	91	Figure 5E
MYC	65	Figure 6A

Knockdown efficiency was calculated as (1 − protein level in siRNA-treated cells/protein level in control cells) × 100%. Data are from three independent experiments.
